# Fast and accurate vision-based stereo reconstruction and motion estimation for image-guided liver surgery

**DOI:** 10.1049/htl.2018.5071

**Published:** 2018-10-19

**Authors:** Andrew D. Speers, Burton Ma, William R. Jarnagin, Sharifa Himidan, Amber L. Simpson, Richard P. Wildes

**Affiliations:** 1 Department of Electrical Engineering and Computer Science, York University, Toronto, ON, Canada; 2 Hepatopancreatobiliary Service, Department of Surgery, Memorial Sloan Kettering Cancer Center, New York, NY, USA; 3 Department of Surgery, The Hospital for Sick Children, Toronto, ON, Canada; 4 Department of Surgery, University of Toronto, Toronto, ON, Canada

**Keywords:** phantoms, image matching, stereo image processing, image reconstruction, computerised tomography, tumours, motion estimation, liver, image registration, medical image processing, surgery, cancer, image texture, time series, accurate vision-based stereo reconstruction, motion estimation, image-guided liver surgery, ablation, adjacent complex vasculature, end-to-end solution, coarse-to-fine stereo approach, liver imaging, three-dimensional boundary recovery, robust 3D motion estimator, adaptive CTF matching approach, liver phantom, precise quantitative evaluation, liver resection, fast vision-based stereo reconstruction, oncologic outcome, low texture regions, adaptive windows, time series, volumetric computed tomography scan, tumours

## Abstract

Image-guided liver surgery aims to enhance the precision of resection and ablation by providing fast localisation of tumours and adjacent complex vasculature to improve oncologic outcome. This Letter presents a novel end-to-end solution for fast stereo reconstruction and motion estimation that demonstrates high accuracy with phantom and clinical data. The authors’ computationally efficient coarse-to-fine (CTF) stereo approach facilitates liver imaging by accounting for low texture regions, enabling precise three-dimensional (3D) boundary recovery through the use of adaptive windows and utilising a robust 3D motion estimator to reject spurious data. To the best of their knowledge, theirs is the only adaptive CTF matching approach to reconstruction and motion estimation that registers time series of reconstructions to a single key frame for registration to a volumetric computed tomography scan. The system is evaluated empirically in controlled laboratory experiments with a liver phantom and motorised stages for precise quantitative evaluation. Additional evaluation is provided through testing with patient data during liver resection.

## 1 Introduction

Liver resection is the only potentially curative therapy for liver cancer but often represents a surgical challenge due to the location of tumours throughout complex, delicate vasculature [1, 2]. Image-guided surgical techniques improve intraoperative localisation [3]. While image guidance has become the standard of care in neurosurgery, the lack of non-rigid correction and reliance on static intraoperative data has hindered adoption in liver surgery. By imaging the liver in real time and providing the surgeon with continuous feedback regarding the localisation of tumours relative to adjacent major vasculature, the likelihood of an incomplete resection or inadvertent liver injury should be minimised.

A variety of approaches have been proposed to acquire real-time intraoperative data, typically in isolation. Intraoperative three-dimensional (3D) structure recovery that relies on monocular imagery has been explored but is often susceptible to error and suffers scale ambiguities without the presence of external markers [4–7]. Instead of directly computing 3D structure from monocular imagery, one approach has been explored that frames the problem of overlaying preoperative volumetric information onto the intraoperative 2D video as that of computing a projection matrix from 2D–3D correspondences between the video sequence and preoperative computed tomography (CT) [8]. Reconstruction from stereo imagery using so-called local [9, 10], semi-global [11, 12] and global approaches [13–17] also has been considered, where the main differences can be viewed as trade-offs between computational efficiency and the complexity of smoothness integration employed in image matching. The previous work has also considered a visual odometry-based approach, making use of the quadrifocal constraints to estimate binocular laparoscopic camera motion in a surgical environment [18]. Stereo imaging has emerged as a promising modality for acquiring rich continuous intraoperative surface data in neurosurgery [19, 20] but a number of challenges need to be solved for such systems to perform adequately in liver surgery. Among the most pressing and immediate challenges are that stereo reconstruction in the liver is non-trivial: the liver is a sparsely textured organ with few features to drive reconstruction algorithms and specular reflections further compromise reconstruction accuracy.

Existing systems for optical organ registration in surgery have been proposed; however, they typically rely on techniques that may ultimately hinder their adoption in soft tissue surgery. Some deployed systems benefit from having a rigid reference frame (e.g. neurosurgery) and assume relatively little deformation of the target organ with respect to this reference frame [21, 22]. Other more recent research has considered automated rigid registration of a liver surface model with a preoperative CT scan by matching shape-based feature descriptors [23]. As rigid constraints are not always applicable to soft tissue surgery, the use of cross-modality fiducials has been proposed for use on the body [24, 25] and on the organ itself [26]. The use of fiducials creates additional invasive steps in the workflow of typical procedures to the degree that it is often desirable to avoid their use altogether. Furthermore, their utility often diminishes greatly as their placement becomes separated from the surgical surface of interest. Other systems operate through the use of manually chosen feature points on the visible anatomy [27]. The reliance on the manual selection and tracing of points in the image sequence is undesirable as it places a barrier for the autonomous operation of the system as a whole. Arguably, the most applicable approach to date is the use of intraoperative cone-beam CT (CBCT) as a bridging modality [28]. The use of CBCT allows for the use of a non-rigid biomechanically driven registration technique [29] for aligning the preoperative CT and intraoperative CBCT scans which allows for the system to compensate for the large non-rigid deformation between the two sources of data. However, the use of CBCT exposes the patient (and surgical team) to repeated doses of radiation to provide this reference.

In the light of previous research, the primary contribution of this work is a novel end-to-end system for fast surface reconstruction and motion estimation for alignment with a preoperative CT scan. Specifically, we deem this system to be ‘end-to-end’ as the proposed system utilises manual input for initialisation purposes only and requires neither human intervention nor does it rely on any intraoperative bridging modalities [such as open magnetic resonance imaging (MRI), CBCT etc.] during subsequent operation. The current instantiation of the system is designed for use during intraoperative planning/exploratory phases. Extensions are underway to make the system applicable for larger portions of the surgical workflow.

Within this end-to-end system, we make the following three sub-contributions. (i) To the best of our knowledge, we are the first to use an adaptive coarse-to-fine (CTF) stereo algorithm for fast and accurate 3D surface reconstruction in intraoperative imaging. The approach yields data-driven dense reconstruction by allowing coarse resolution image data to inform fine resolution reconstruction, even in low texture regions [30]. CTF processing also leads to computational efficiency, while the complementary use of adaptive windows supports the precise reconstruction of 3D boundaries. (ii) We make use of a robust, 3D motion estimator based on interframe feature matching to register a time series of reconstructions to a single key frame for registration to a volumetric CT scan. Unlike most approaches that use the iterative closest point algorithm for 3D model registration, our feature matching-based approach ensures that registration brings physically meaningful features into alignment and does so without chaining multiple incremental registrations that can rapidly lead to drift. (iii) A mask denoting the boundary of the organ of interest, the liver, is automatically maintained within the system. This has been studied in a standalone fashion (e.g. [31]) but the use of such information within the context of this system raises new insight. Maintaining a mask of the liver boundary not only allows for efficient processing of the information by restricting processing to the portion of the video stream imaging the liver but also allows for the system to take advantage of the fact that the intraoperative motion of the liver during exploratory phases is predominantly rigid, allowing for more robust motion estimation.

The system has been evaluated empirically in controlled laboratory experiments with a liver phantom placed on motorised stages for precise quantitative evaluation. Our phantom-based datasets are available to the research community and can be found at http://vision.eecs.yorku.ca/research/medical/. Additional evaluation has been undertaken with clinical data. Both evaluations take place in open liver resection conditions. Notably, while much research effort focuses strictly on laparoscopic surgeries, most liver resections are performed as open resections due to the extent and location of disease. In our centre, roughly 75% of liver resections are performed in an open environment [32]. Moreover, laparoscopic approaches are only suitable for certain cases where there is oncologic equivalency between open and laparoscopic [32]. Overall, we demonstrate a clinical stereo-based platform capable of reliably providing temporally dense 3D textured data in near real-time under realistic conditions of liver surgery.

Interestingly, the only two Food and Drug Administration (FDA)-approved liver surgery systems do not compensate for real-time motion [33]. More generally, commercial systems rely on optical tracking, which has a stated accuracy of <2 mm, as evaluated at a single point in time [34]. The errors we report are within comparable bounds and our system is capable of providing continual updates. Thus, our approach has potential to provide the precise anatomical location of tumours within the complex vasculature, in real-time, as the liver undergoes motion throughout the course of surgery.

## 2 Technical approach

**Fig. 1:**
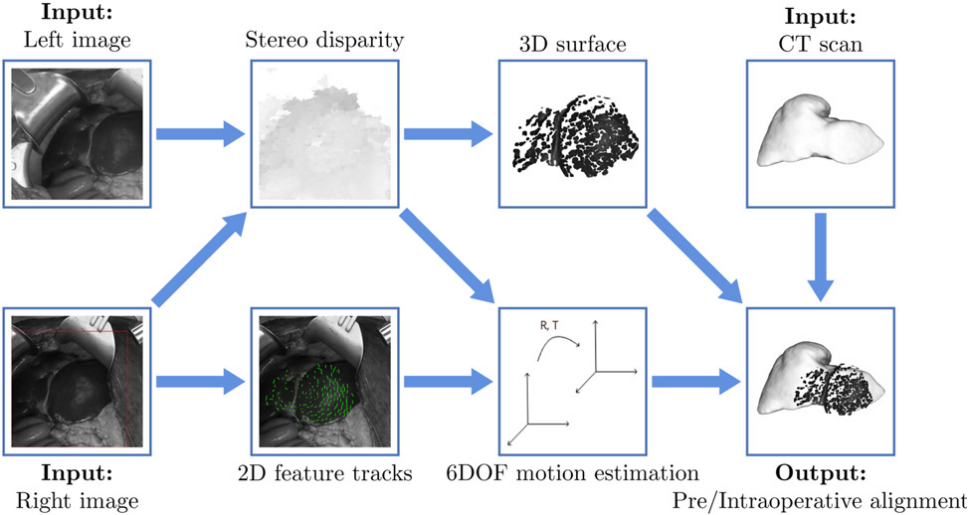
System diagram. Input images from stereo video cameras are shown in grey level with a red box indicating the region of analysis. Recovered stereo correspondences are shown as a disparity map referenced to the right image, with brighter grey levels indicating larger disparities. Green lines on tracking output show recovered 2D displacement of features across time. Pre/intraoperative alignment shows an overlay of the reconstructed surface map on the volumetric model

Fig. 1 provides an overview of the system for recovering a time series of 3D surface reconstructions of a surgical scene and registering to a preoperative volumetric model. The input is a pair of synchronised images from a stereo video camera (left and right images) and a volumetric model (CT scan). The processing pipeline consists of three main components: stereo correspondence determination, 2D feature tracking and six degrees of freedom (6DOF) motion estimation. Stereo correspondence yields a dense disparity map between points in the left and right images. The disparity map is projected into 3D space and filtered to produce a 3D surface reconstruction. 2D feature tracking is applied to the video from the right camera to provide 2D matched feature locations across the image sequence. These 2D tracks are fused with the disparity maps to produce a 3D non-rigid deformation field. 6DOF motion estimation regresses the 3D deformation field to a rigid 6DOF motion relating the current frame back to a key frame. The 3D surface reconstruction and 6DOF motion estimate are combined to place the surface into the same reference frame as a key frame. When combined with a hand registration of the key frame to the preoperative scan, the registration of incoming frames back to the key frame allows for a chained registration back to the preoperative volumetric scan of the organ of interest.

### 2.1 Stereo correspondence and 3D surface reconstruction

The system uses a local stereo correspondence algorithm that has been shown to provide accurate and efficient depth estimates [30]. Given a calibrated stereo pair of images, ${\bi I}_l\lpar x\comma \; \, y\rpar $ and ${\bi I}_r\lpar x\comma \; \, y\rpar $, the algorithm yields a disparity map, ${\bi d}\lpar x\comma \; \, y\rpar $, that provides the spatial offset between corresponding points in the input pair. The disparity map is recovered by solving the optimisation problem

(1) $${\bi d}\lpar x\comma \; \, y\rpar = \mathop {\arg \max }\limits_{{d}_i \in D} \sum\limits_{\left({u\comma \, v} \right)\in w\left({x\comma \, y} \right)} \rho \left[{{\bi I}_l\lpar u\comma \; \, v\rpar \comma \; {\bi I}_r\lpar u + {d}_i\comma \; \, v\rpar } \right]\comma \; \eqno\lpar 1\rpar $$

via search over disparities, $d_i$, in a specified range, *D*, to maximise the summed pixel-wise similarity measure, $\rho $, between image intensity values within a window, *w*, around $\lpar x\comma \; \, y\rpar $. Although the algorithm does not rely on the choice of a specific similarity measure, $\rho $, our instantiation makes use of normalised cross-correlation. The algorithm employs CTF processing for efficiency, whereby initial low-resolution versions of the input images yield low-resolution disparity maps that subsequently are refined via consideration of higher-resolution images to culminate with the resolution of the original input. The algorithm also uses adaptive windows, *w*, that confirm to avoid smoothing across 3D boundaries. An example recovered disparity map for an input stereo pair is shown in Fig. 1.

Use of known camera calibration allows for the recovered disparities to be back-projected to a 3D point cloud. The resulting point cloud is filtered via statistical outlier removal to reject depth values that differ significantly from their neighbours [35] as well as resampled via moving least squares [36] to produce a smooth manifold surface as the final 3D surface reconstruction. A manually specified region of interest mask can be provided for an initial (key) frame in the image sequence to restrict processing to the area to be registered to the volumetric model. Following initial delineation, the mask is warped automatically to all other frames in the sequence via a robust affine estimate of image motion across the masked region [37]. An example of recovered surface reconstruction is shown in Fig. 1.

### 2.2 2D feature tracking and generation of a 3D deformation field

To establish feature tracks between frames in the stereo video sequence, 2D features are tracked in one of the video streams (the right is used) and subsequently are projected to 3D. Tracking in 2D makes use of the ‘good features to track’ algorithm [38]. This algorithm restricts operations to feature points, $\lpar x\comma \; \, y\rpar $, where the local image gradient structure is sufficient for stable appearance across time. Tracking is performed on the extracted features across two images, ${\bi I}^t$ and ${\bi I}^{t + \delta t}$, taken at times *t* and $t + \delta t$, respectively, by minimising the dissimilarity measure

(2) $$\sum\limits_{\left({u\comma \, v} \right)\in r\left({x\comma \, y} \right)} [{{\bi I}^{t + \delta t}\lpar u + \delta _x\comma \; \, v + \delta _y\rpar - {\bi I}^t\lpar u\comma \; \, v\rpar } ]^2\comma\eqno\lpar 2\rpar $$

over windows, *r*, centred at the feature points. A gradient-based solution is employed to yield the optimal feature displacement, $({\delta _x\comma \; \, \delta _y} )$, for each feature point, $\lpar x\comma \; \, y\rpar $. While the original formulation accounted for a full affine transformation across time [38], the simpler translational formulation given here has proved to suffice for the cases of current interest. Example tracks are shown in Fig. 1.

Analogous to the back-projection of stereo disparity maps to 3D point clouds (Section 2.1), the extracted feature tracks are combined with the disparity estimates at the tracked points to yield 3D deformation fields. These fields provide a sequence of 3D feature correspondences between all frames in the sequence, capturing both the rigid and non-rigid components of the motion of the tracked region in 3D.

### 2.3 6DOF motion estimation and pre/intraoperative alignment

Alignment between the time sequence of 3D surface reconstructions and the volumetric model is initialised via manual registration of a key reference frame in the reconstructions and the model. This registration is given as a 6DOF rigid transformation specified via a graphical user interface. (Future work will refine the initial alignment with an automated non-rigid registration algorithm.) Given this initial registration, all subsequent surface reconstructions from the stereo video sequence are registered to the first frame to inherit its alignment to the volumetric model.

To perform the registration across frames of the 3D surface reconstructions, a 6DOF motion between each frame and the key frame is recovered based on the previously recovered 3D deformation fields (Section 2.2). (To complement future work that considers a non-rigid initialisation to the volumetric model, the non-rigid residual to the 6DOF estimate can be considered.) The rigid transformation is recovered by a robust version of an earlier algorithm according to

(3) $$\left({{\bi R}\comma \; \, {\bi t}} \right)= \mathop {\arg \min }\limits_{{\bi R} \in {\rm SO\lpar }3\rpar \comma {\bi t} \in {\opf R}^3} \sum\limits_{i = 1}^n \Vert\left({{\bi R}{\bi p}_i + {\bi t}} \right)- {\bi q}_i\Vert^2\eqno\lpar 3\rpar $$

where ${\bi p}_i$ and ${\bi q}_i$ are 3D points in two surface reconstructions that have been brought into correspondence at *n* locations tracked by the 3D deformation field, ***R*** is a $3 \times 3$ rotation matrix and ${\bi t}$ is a $3 \times 1$ translation vector. The rotation is found using singular value decomposition on the covariance matrix relating the two point sets after placing their centroids at the origins (${\bi {\,p}^{\prime}}$ and ${\bi {q}^{\prime}}$). The optimal translation is then the residual created in the origin-centric point clouds after the rotation is applied (i.e. ${\bi t} = {\bi {q}^{\prime}} - {\bi R}{\bi {\,p}^{\prime}}$). The solution is made robust using the random sample consensus algorithm to minimise the effect of outlier correspondences or those most affected by any non-rigidity. Notably, registering incoming frames back to a key frame ameliorates issues of registration drift that can occur in the alternative approach of chaining sequential registrations between adjacent frames over a long sequence.

An example of final alignment between a 3D surface reconstruction and volumetric model is shown in Fig. 1. For input $640 \times 480$ images, the system executes at 8 fps on a 3.6 GHz processor. Considerable speed up is anticipated with a graphics processing unit (GPU) implementation, e.g. the slowest component of the system is stereo correspondence, which already has been ported to run on a 512 core GPU (nVIDIA GeForce GTX580) at 100 fps.

## 3 Empirical evaluation

**Fig. 2:**
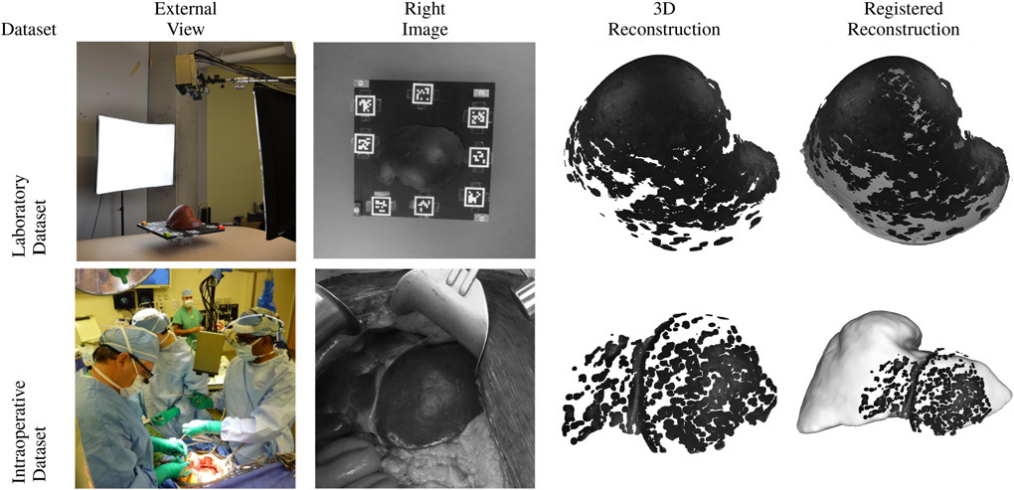
Overview of experiments

**Fig. 3:**
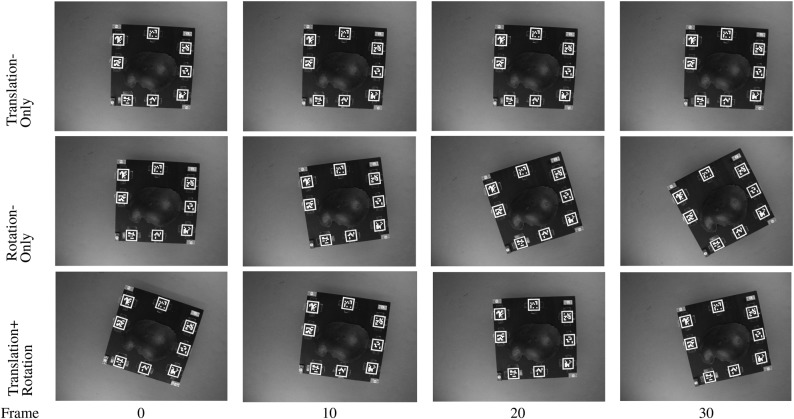
Laboratory datasets

The proposed system was evaluated empirically in both controlled laboratory conditions and with clinical data acquired during liver resection. Fig. 2 shows images from the test scenarios as well as acquired images, surface reconstructions and final registrations for both laboratory and operating room conditions. The laboratory dataset, depicted in Fig. 3, was acquired using a silicone liver phantom. The phantom was rigidly affixed to a motion control platform (one linear and one rotational stage – Newport Corporation, Irvine, CA) that allowed for the phantom to undergo precise motion patterns. The platform contains cross-modality features used for alignment of the initial frame to a CT scan. Each laboratory test condition consisted of a 31 frame sequences with evenly spaced samples along the motion trajectory. Three different motion profiles were tested: translation-only (1 mm increments), rotation-only (1° increments) and translation + rotation motions superimposed. The laboratory dataset was acquired at a standoff distance of 700 mm to the liver (similar to the target distance for the device used intraoperatively). The intraoperative data sequence was taken during an open liver resection and consisted of 100 frames. In both the laboratory and operating rooms, a calibrated stereo video camera was used for image acquisition (the gold box in the upper middle portion of the external views shown in Fig. 2).

**Fig. 4:**
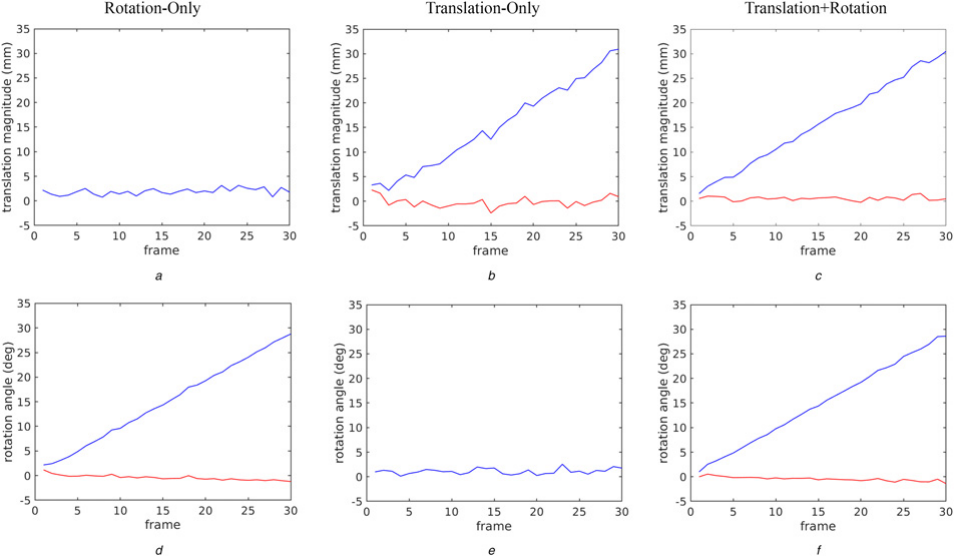
Motion recovery results. The recovered translation magnitude (a–c) and rotation (d–f) estimates are plotted for each of the three laboratory motion profiles (rotation-only, translation-only and translation + rotation). The blue line indicates the motion estimate; the red line depicts the deviation of the motion estimate from the ground truth, as actuated by the motion platform. The two plots showing a single curve correspond to measurements, where the ground truth signal was zero motion; hence, the recovered and error values are the same and only the recovered is shown *a–c* Recovered translation magnitude *d–f* Recovered rotation

The translation (magnitude) and rotation components of the recovered motions are reported for the laboratory datasets in Fig. 4. For each of the three tested motion profiles (rotation-only, translation-only and translation + rotation), the recovered translation magnitude is shown in Figs. 4*a–c*, while the recovered rotation angles are shown in Figs. 4*d–f*. At any given frame, the motion was recovered with respect to the initial frame in the sequence. For rotation-only, it is seen that the recovered angle accurately tracks the true motion (interframe increments of 1°), approximately a line of slope one and the recovered translation is correctly very small. (Note that since the true translation has zero magnitude, the error between the recovered and ground truth is the same as the recovered and only the recovered is shown.) For the translation-only case, the results are exactly complementary to those of rotation-only, again showing very accurate performance. Similarly, the translation + rotation shows the desired combination of the other two cases. Notably, motion drift is not affecting the interframe estimation over these sequences owing to the motion always being computed relative to the initial frame. In contrast, typical approaches that chain transformation estimates between adjacent frames to relate a given frame back to the key frame would be susceptible to drift.

**Fig. 5:**
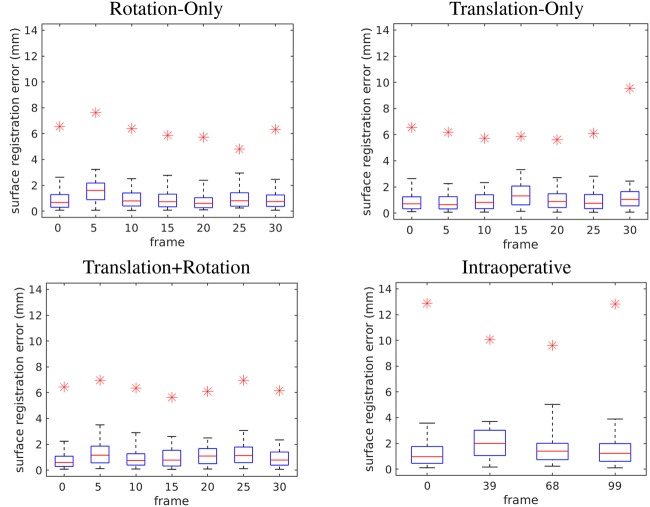
SRE (4) is shown at 5°/mm increments across the laboratory sequences. For the intraoperative data, four frames representing various amounts of respiratory motion were chosen to show the system performance over the largest portion of the organ's motion. Box plots are shown for each of the reported frames. Whiskers cover 95% of the reconstructed point clouds. The largest outlier for each dataset is indicated by a red asterisk

A measure of surface registration error (SRE)

(4) $${\rm SR}{\rm E}_i = \Vert{\bi p}_i - {\bi p}_i^\ast \Vert\comma \; \eqno\lpar 4\rpar $$

where ${\bi p}_i$ is a point in the registered stereo reconstruction of the surface and ${\bi p}_i^\ast $ is the closest point to ${\bi p}_i$ in the preoperative volume, was calculated for all laboratory and intraoperative datasets. The plots show the distribution of SRE over all points in the reconstructed point clouds for the selected frames. Registration between CT coordinates and the initial frame of each laboratory sequence was performed in a semi-automated manner, via identification of five cross-modality features located on the platform supporting the phantom in both the stereo imagery and in the CT scan. A similar process was used for the intraoperative data after an initial hand alignment of the datasets. This procedure defines the transformation relating the initial (key) frame to the CT coordinate space. Subsequent frames were registered to the initial key frame using the approach described in Section 2.3 and then registered to CT space using the transformation relating the key frame to the CT scan. Fig. 5 shows SRE for the three laboratory datasets and the intraoperative dataset. Median errors are typically on the order of 1 mm with 95% of the points lying under 4 mm SRE across the reported datasets. Notably, the interframe registration algorithm never explicitly minimises SRE by applying shape-based registration techniques (e.g. iterative closest point); therefore, the registration is data driven with respect to the actual positions of identified features on the surface of the organ and is more likely to produce a physically meaningful registration.

Table 1 provides a summary of the average and standard deviation of the reported SRE and motion errors for the three laboratory datasets and the intraoperative dataset. Note that average errors and standard deviation of the motion estimates are not provided for the intraoperative dataset as no ground truth motion information is available.

**Table 1:** Mean and standard deviation of reported errors

Dataset	SRE, mm		Translation magnitude, mm		Rotation angle, deg	
	Avg	Std	Avg	Std	Avg	Std
rotation-only	1.0274	0.8698	1.8975	0.6547	−0.4124	0.5126
translation-only	1.0597	0.8736	−0.1831	0.9863	1.0898	0.5744
translation + rotation	1.0785	0.8998	0.5656	0.4092	−0.4507	0.4095
intraoperative	1.6493	1.4060	n/a	n/a	n/a	n/a

## 4 Conclusion

We have presented an end-to-end system for fast and accurate 3D surface reconstruction and motion estimation for alignment with a preoperative volumetric scan. Key technical innovations include the use of an adaptive CTF algorithm for efficient and accurate 3D surface reconstruction from stereo imagery and use of a robust, feature-based 3D motion estimator for physically meaningful alignment. The system has been evaluated both quantitatively and qualitatively in controlled laboratory conditions as well as with clinical data. The results suggest the potential for integration into a clinical system. Future work will make further use of the recovered 3D deformation field (Section 2.2) to support non-rigid refinements for our 3D surface reconstructions to volumetric model alignments. An extension of this approach to include subsurface registration through the use of additional sensing modalities that support subsurface data acquisition during surgery (e.g. ultrasound, CBCT and open MRI) is also of interest. Appropriate application of intraoperative subsurface scanning and registration constraints may provide better localisation for surgeons when surgical margins are tight. Furthermore, integrating subsurface information into the surface-based registration approach is an important step for being able to measure the correlation between measurements of registration errors at the surface of the organ and the ability to target subsurface structures accurately. Finally, additional testing on clinical data is desired in order to further validate and develop these techniques.

## 5 Funding and declaration of interests

This research was supported in part by the Health Ecosphere as funded by the FedDev Ontario (807351), the Big Data Research and Analytics Information Network (BRAIN) as funded by the Ontario Research Fund (RE07-023) and the NIH/NCI Cancer Center Support Grant no. P30 CA008748.

## 6 Conflict of interest

None declared.
